# On the rate of phytoplankton respiration in the light

**DOI:** 10.1093/plphys/kiac254

**Published:** 2022-06-02

**Authors:** Michael L Bender, Xin-Guang Zhu, Paul Falkowski, Fangfang Ma, Kevin Griffin

**Affiliations:** School of Oceanography, Shanghai Jiao Tong University, Shanghai 200030, China; Department of Geosciences, Princeton University, Princeton, New Jersey 08544, USA; State Key Laboratory of Plant Molecular Genetics, Center of Excellence for Molecular Plant Sciences, Chinese Academy of Sciences, Shanghai 200032, China; Environmental Biophysics and Molecular Ecology Program, Department of Marine and Coastal Sciences, Rutgers, The State University of New Jersey, New Brunswick, New Jersey 08901, USA; State Key Laboratory of Crop Biology, College of Horticulture Science and Engineering, Shandong Agricultural University, Tai-An, Shandong 271018, China; Department of Earth and Environmental Sciences, Columbia University, Palisades, New York 10964, USA; Department of Ecology, Evolution and Environmental Biology, Columbia University, New York, New York 10027, USA; Lamont-Doherty Earth Observatory, Columbia University, Palisades, New York 10964, USA

## Abstract

The rate of algal and cyanobacterial respiration in the light is an important ecophysiological term that remains to be completely characterized and quantified. To address this issue, we exploited process-specific decarboxylation rates from flux balance analysis and isotopically nonstationary metabolic flux analysis. Our study, based on published data, suggested that decarboxylation is about 22% of net CO_2_ assimilation when the tricarboxylic acid cycle is completely open (characterized by the commitment of alpha ketoglutarate to amino acid synthesis and very low rates of succinate formation). This estimate was supported by calculating the decarboxylation rates required to synthesize the major components of biomass (proteins, lipids, and carbohydrates) at their typical abundance. Of the 22 CO_2_ molecules produced by decarboxylation (normalized to net assimilation = 100), approximately 13 were from pyruvate and 3 were from isocitrate. The remaining six units of decarboxylation were in the amino acid synthesis pathways outside the tricarboxylic acid cycle. A small additional flux came from photorespiration, decarboxylations of six phosphogluconate in the oxidative pentose phosphate pathway, and decarboxylations in the syntheses of lower-abundance compounds, including pigments and ribonucleic acids. This general approach accounted for the high decarboxylation rates in algae and cyanobacteria compared to terrestrial plants. It prompts a simple speculation for the origin of the Kok effect and helps constrain the photoautotrophic respiration rate, in the light, in the euphotic zone of the ocean and lakes.

## Teaser

In the light, net nonphotorespiratory decarboxylations are generally associated with biosynthesis and sum to about 25% of net carbon assimilation according to two independent approaches.

## Introduction

Respiration generates energy and provides substrates for biosynthesis. In photoautotrophs, understanding decarboxylation rates in the light involves identifying the individual metabolic pathways that produce CO_2_, constraining the decarboxylation rate associated with each pathway, and quantifying the cellular decarboxylation rate as the sum of the rates associated with the individual decarboxylating processes.

Decarboxylation in the light (“day respiration”) has been a topic of active research going back over 70 years (Kok, 1949, 1956; Tcherkez et al., 2017b; Tcherkez and Atkin, 2021). However, basic questions remain about the roles of the different processes and pathways. Progress was slow because the decarboxylation rate in the light is hard to measure accurately, and there were few measurements of the decarboxylation rates associated with the specific biochemical pathways. Over the last decade, this situation has changed with the publication of rates in the intermediate metabolism constrained by flux balance analysis (FBA) and isotopically nonstationary metabolic flux analysis (INST-MFA) (Young et al., 2011; Kim et al., 2016;  Treves et al., 2022) and citations below. For cultured phytoplankton (aquatic algae and cyanobacteria), we now have available carboxylation rates by ribulose-1,5-bisphosphate carboxylase-oxygenase (RuBisCo) and phosphoenolpyruvate (PEP) carboxylase. We also know decarboxylation rates of pyruvate (PYR), isocitrate and alpha ketoglutarate (AKG) in the tricarboxylic acid (TCA) cycle, along with malic enzyme (ME), photorespiration, and the oxidative pentose phosphate pathway (OPPP). Also, we can calculate decarboxylation rates associated with amino acid synthesis outside PYR dehydrogenase (PDH) and the TCA cycle (see below and Supplemental Text S2). Thus, we can better characterize decarboxylation in the light, and understand its functions.

Our focus is on “nonphotorespiratory, uncompensated decarboxylations” (NU-decarboxylations). “Uncompensated decarboxylations” exclude processes in which decarboxylations are compensated by carboxylations in closely connected metabolic pathways, leading to zero net flux. NU decarboxylation is a term of merit, because it is equal to the net nonphotorespiratory decarboxylation rate of a cell. As well, it is equal to the decarboxylation rate associated with biosynthetic pathways when the TCA cycle is fully open.

**Figure 1:**
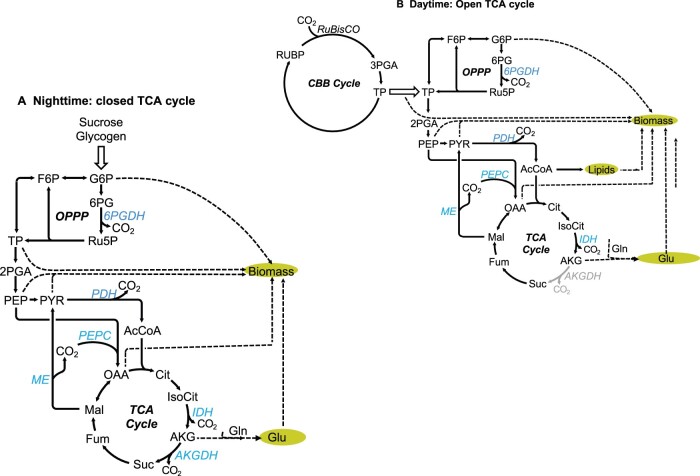
Simplified flux map of the intermediate metabolism. A, Dark (closed TCA cycle). B, Light (open TCA cycle). CBB, Calvin–Benson cycle; RuBP, ribulose 1-5-bisphosphate; PGA, phosphoglycerate; TP, triose phosphate; GAP, glyceraldehyde 3-phosphate; FBP, fructose 1-6 bisphosphate; F6P, fructose-6-phosphate; G6P, glucose-6-phosphate; R5P, ribulose-5-phosphate; 6PG, 6 phosphogluconate; R5P, ribose 5 phosphate; TP, triose phosphate; PEP, phosphoenolpyruvate; Pyr, pyruvate; PDH, pyruvate dehydrogenase; PEPC, PEP carboxylase; AcCoA, acetyl CoA; ME, malic enzyme; OAA, oxaloacetate; Cit, citrate; Isocit, isocitrate; IDH, isocitrate dehydrogenase; AKG, alpha ketoglutarate; AKGDH, AKG dehydrogenase; fum, fumarate; mal, malate.

The hub of decarboxylation reactions in the intermediate metabolism is acetyl Co-A, which is in turn produced by decarboxylation of PYR. Acetyl Co-A may combine with oxaloacetate to enter the TCA cycle as citrate. In the dark, the TCA cycle is “closed” and two citrate carbon atoms undergo decarboxylation, accompanied by the production of reducing equivalents and ATP (Figure 1A). However, in the light (Figure 1B), citrate loses one carbon atom to decarboxylation, and the second can be retained for the formation of glutamate and other amino acids (Hurry et al., 2005; Sweetlove et al., 2010; Tcherkez et al., 2017b). Hence, the TCA cycle can be broken (or open) in the light. In the open TCA cycle, in the light, carbon fluxes are largely or entirely committed to biosynthesis (Figure 1B), although there are still decarboxylations, and some NADH is produced. For example, the synthesis of each molecule of glutamate will be accompanied by the production of two molecules of CO_2_. One is from PYR decarboxylation, and the other is from isocitrate decarboxylation in the TCA cycle. Xu et al. (2021) and Sweetlove et al. (2013) leveraged this linkage to calculate relative decarboxylation rates by the various metabolic pathways in plant tissues and other organisms. This study builds on their approach. We invoke stoichiometric and mass-balance constraints (Penning de Vries et al., 1974) to assess the contributions to decarboxylations that are made in the course of protein, lipid, and carbohydrate synthesis in the light (Supplemental Texts S2 and S3). We tabulate or calculate decarboxylation rates for a critical subset of metabolic processes normalized to net C assimilation = 100 units.

### NU-decarboxylations constrain net nonphotorespiratory CO_2_ release and the decarboxylation rate associated with biosynthesis

Respiration has been defined as the nonphotorespiratory release of CO_2_ (Atkin et al., 1997; Tcherkez et al., 2017b; Xu et al., 2021). For our work, however, this definition is problematic. The reason is that, in the light, large decarboxylation fluxes in the intermediate metabolism may be associated with cyclic or “compensated” processes leading to zero net flux. Offsetting carboxylations and decarboxylations are associated with each step in the elongation of fatty acid chains (Carvalho and Caramujo, 2018). As shown below, ME and PEP carboxylase produce and consume CO_2_ at rates that are linked, and therefore partly offsetting. From a mass balance perspective, part of carboxylation at RuBisCo compensates for decarboxylation in the OPPP.

These offsetting or compensated fluxes are not registered in net CO_2_ assimilation. We therefore focus on NU-decarboxylations. This term excludes photorespiratory CO_2_, carboxylations and decarboxylations associated with each individual step in the elongation of fatty acid chains, PEP carboxylase balanced by the loss of CO_2_ from ME, and pentose phosphate decarboxylations (specifically, 6-phosphogluconate [6-PG] decarboxylations) that consume an equal amount of carbon fixed by RuBisCo. When the TCA cycle is completely open (no decarboxylation of AKG) (Figure 1B), and there is no photorespiration, decarboxylations come almost entirely from PYR and isocitrate, plus decarboxylations in amino acid syntheses outside the TCA cycle. In this case, NU-decarboxylation is the same as biosynthesis decarboxylation (defined as the decarboxylations that are integral to biosynthetic pathways). Certainly, many other decarboxylation reactions are essential to metabolism, but their fluxes are small (e.g. Kim et al., 2016). Examples include decarboxylations during syntheses of ribonucleic acids, chlorophyll, and accessory pigments.

### Two independent approaches constrain the respiration rate in the light

In this paper, we deal exclusively with constraints imposed on decarboxylation in the light by stoichiometry and mass balance. A discussion of enzymatic processes mediating metabolic rates is beyond the scope. For the sake of simplicity, we focus primarily on prokaryotic and eukaryotic phytoplankton rather than land plants, although some governing principles are likely to be similar in both groups of organisms. Phytoplankton have carbon concentrating mechanisms that suppress photorespiration, thereby simplifying the intermediate metabolism. They commit less biomass to carbohydrates and structure with low turnover, thereby elevating the imprint of biosynthesis on the decarboxylation rate (as discussed below). Phytoplankton do not pose challenges associated with the presence of many different forms of tissue. We also simplify by focusing on decarboxylation in the light, rather than relative rates of decarboxylation in light and dark.

We use two approaches to evaluate NU-decarboxylation fluxes. First, we extract decarboxylation rates for the intermediate metabolism derived from published FBA and INST-MFA studies in the literature. These reveal the origin and importance of decarboxylations in the light. From these rates, we calculate gross C assimilation, total decarboxylation, net C assimilation, and the ratio of NU-decarboxylation/net C assimilation.

The second approach invokes the composition of biomass as a constraint on net assimilation and NU-decarboxylation. In this approach, we use published concentrations of the major compounds or compound classes (terms used interchangeably here) that comprise most of the biomass of algae and cyanobacteria. We focus on amino acids, lipids, and carbohydrates, which together comprise about 90% of biomass (Finkel et al., 2016; Liefer et al., 2019). From data on the abundance and composition of the compound classes, we calculate the number of C atoms in biomass (i.e. net assimilation). We also calculate the number of decarboxylations required to synthesize the observed abundance of each of these compound classes (Table 1 and Supplemental Text S3). The sum of decarboxylations required for the syntheses of all the compound classes approximates NU-decarboxylation. We then calculate the ratio of NU-decarboxylation to net C assimilation.

**Table 1:** Moles of C in 100 g of each compound class, decarboxylations required to produce 100 g of each compound class, and decarboxylations required for the production of each compound class normalized to net C production

Compound class	Net production, moles C/100 g of compound	NU-decarboxylations, mole CO_2_/100 g of compound (net production)	NU-decarboxylations/net production
Amino acids	3.78	0.8	0.21
Fatty acids	7.14	3.57	0.5
Carbohydrates	3.33	0	0
Carotenoids (isoprene)	7.36	5.89	0.8
Chlorophyll	6.16	1.79	0.29
DNA + RNA	3.08	0.47	0.15

*Notes*: “NU-decarboxylations” are nonphotorespiratory decarboxylations uncompensated by carboxylations in a related biochemical process.

As a simple example, we calculate the number of decarboxylations that will accompany the production of average phytoplankton biomass as estimated from the compilation of Jonasdottir (2019). In the average sample, 100 g of this biomass contains 51 g of protein, 20 g of lipids, and 29 g of carbohydrate. Using the values in Table 1, these abundances correspond to 1.928 moles of amino C, 1.428 moles of fatty acid C, and 0.966 moles of carbohydrate C per 100 g of biomass. Net assimilation is the sum of these three numbers, 4.320 moles C/100 g of biomass. According to data in Table 1, synthesis of 51 g of protein requires decarboxylation producing 0.409 moles of CO_2_, and synthesis of 20 g of lipids requires decarboxylation of 0.714 moles. Synthesis of 29 g of carbohydrate C does not lead to NU-decarboxylation. Total NU-decarboxylation is thus 0.409 + 0.714 = 1.123 moles. The ratio of NU-decarboxylation to net C assimilation, 1.123 moles/4.320 moles, is 0.26. This value is similar to values obtained from FBA and INST-MFA as outlined above (0.22 ± 0.02).

### In the light, most decarboxylations are associated with a small number of processes linked to biosynthesis

Three sets of reactions are responsible for most NU-decarboxylations of phytoplankton in the light (see, e.g. FBA and INST MFA experiments summarized below). The first is decarboxylation of PYR to make acetyl Co-A. Acetyl Co-A can either combine with oxaloacetate to form citrate or lengthen fatty acid chains. The second is decarboxylation via isocitrate dehydrogenase (ICDH) to make AKG. The third is decarboxylations that take place outside of PDH and the TCA cycle, i.e. in the standard pathways of amino acid synthesis.

In phytoplankton, decarboxylations in the light are linked to the biosynthesis of proteins and lipids, and to a lesser extent ribonucleic acids, chlorophyll, and carotenoids (used here to represent accessory pigments). Based on the stoichiometric relationships and mass balance in the metabolic pathways, we calculate the number of decarboxylations required for the synthesis of 100 g of amino acids, lipids, and carbohydrates (Table 1). We include the number of decarboxylations associated with the synthesis of the proteinogenic amino acids outside of PDH and the TCA cycle, which represents an important flux. We do not track other compound classes such as ribonucleic acids, chlorophyll, and carotenoids, both because of their low abundance and the paucity of data. As we show below, the resulting error is very small.

Except for the OPPP, the reactions involving carbohydrates do not lead to decarboxylations in the light (Sweetlove et al., 2010; Kim et al., 2016; Xu et al., 2021). Of course, oxidation of carbohydrates involves decarboxylations, but the rates in the light are small or zero in most FBA and INST-MFA studies. However, the production of carbohydrates dilutes the concentrations of other metabolites, per unit of biomass, whose synthesis pathways do involve decarboxylations. The effect of carbohydrate synthesis is thus to decrease the decarboxylation rate in the light per unit biomass. Therefore, we track mass fluxes of carbohydrates along with the other compound classes.

### We identify compensated carboxylations and decarboxylations, and then calculate NU-decarboxylations using fluxes from FBA and INST-MFA

**Figure 2:**
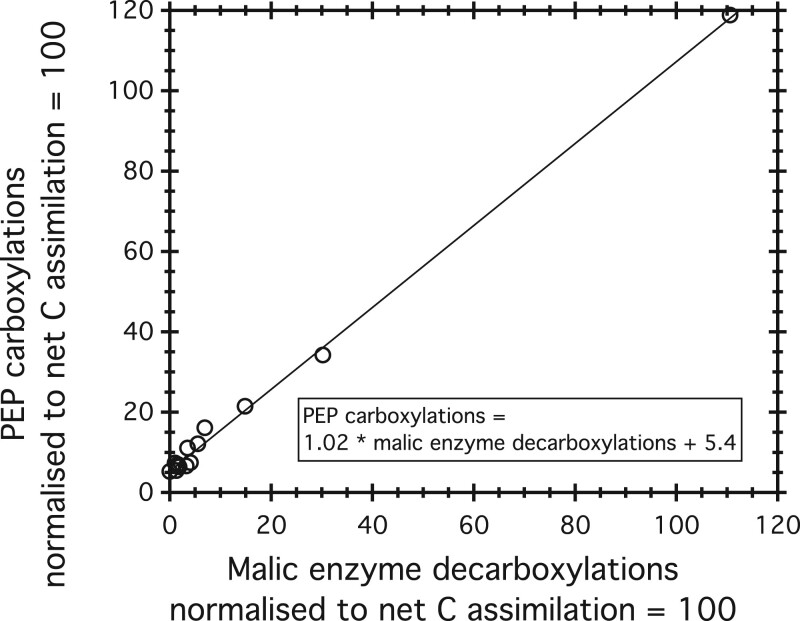
PEP C carboxylation rate versus ME decarboxylation rate for all experiments analyzed in this work. Rates for both PEP carboxylations and ME decarboxylations are normalized to a rate of 100 for net C assimilation. The results fall along a line with slope very close to 1. This result shows that ME and PEP C compensate, resulting in zero net carbon flux, except for a roughly constant rate of about 5% of net C assimilation reflected in the *y*-axis intercept.

To calculate NU-decarboxylation, we start with the observation (Figure 2) that the decarboxylation of malate via ME, as calculated from FBA models and INST-MFA data, varies with PEP C (PEP carboxylase) carboxylations according to the equation

(1) $$\text{PEP}\;C\;\text{carboxylations}=1.02\;*\;\text{ME decarboxylations}+5.4;r^{2}=0.998.$$

The intercept, 5.5 atoms/100 C atoms net assimilation, reflects the rate of the anapleurotic reaction in the absence of ME. This ratio of PEPC/net assimilation is fixed by the stoichiometric requirement of feeding oxaloacetate into the TCA cycle and the synthesis of other metabolites (Falkowski and Raven, 2007). The slope near unity, and the high *r*^2^, suggests the robust covariation of PEP C and ME, although confidence should be tempered by the small data set and the strong influence of high-flux samples. In our accounting, PEP production via PEP carboxylase is compensated with respect to CO_2_ by ME decarboxylations when the PEP C carboxylation rate exceeds 5.4 C atoms per 100 atoms of net C assimilation. Evidence for a partly compensated process, with no net CO_2_ fluxes comes, for example, from Qian et al. (2018). They conclude that PYR is produced from PEP by the sequence: PEP + CO_2_ → OAA → MAL → PYR + CO_2_ (OAA is oxaloacetate and MAL is malate).

Decarboxylation fluxes of 6-PG (Figure 1 and Table 2) produce reducing equivalents that may be large, and important for cellular metabolism (Buckley and Adams, 2011; Qian et al., 2018; Xu et al., 2021). From a mass-balance perspective, we consider OPPP decarboxylations as part of a cycle in which decarboxylations of 6-PG are compensated by a fraction of the carboxylation flux of RuBisCo. An example is the cycle: RuBP + CO_2_ → 2 3PG → 2 GAP → FBP → F6P → G6P → 6PG → R5P + CO_2_ → Ru5P + CO_2_ → RuBP + CO_2_ (Qian et al., 2018) (RuBP, ribulose 1-5-bisphosphate; 3-PG, 3-phosphoglycerate; GAP, glyceraldehyde 3-phosphate; FBP, fructose 1-6 bisphosphate; F6P, fructose-6-phosphate; G6P, glucose-6-phosphate; and R5P, ribulose-5-phosphate). In this context, 6-PG decarboxylation is part of a cycle with NU-decarboxylation flux = zero. This treatment of the OPPP is somewhat counterintuitive. However, it is internally consistent and useful as an accounting tool. It is also justified by the fact that the 5-C product of decarboxylation (R5P) is transformed to become the substrate for carboxylation (RuBP). In any case, OPPP decarboxylations were negligible in most FBA and INST-MFA experiments (Table 2).

**Table 2:** Fluxes in two experiments with completely open TCA cycle (Kim et al., 2016; Jazmin et al., 2017), three experiments with partly closed TCA cycle (Young et al., 2011; Qian et al., 2018), and three experiments with plants (Ma et al., 2014; Xu et al., 2021)

	Results of FBA and INST-MFA fluxes with the TCA cycle completely open		INST-MFA, partly closed TCA cycle			INST-MFA, plants		
**Citation**	Jazmin et al., 2017	Kim et al., 2016	Qian et al., 2018	Qian et al., 2018	Nakajima et al., 2017	Ma et al., 2014	Ma et al., 2014	Xu et al., 2021
Taxon	*Synechococcus elongatus* PCC 7942	*Phaeodactylum tricornutum*	*Synechococcus* 7002	*Synechococcus* 7002	Glucose-tolerant Synechocystis PCC 6803	*Arabidopsis thaliana*	*Arabidopsis thaliana*	*Camelina*
Irradiance (µmol m^−2^ s^−1^)	150	150-200	60	60	120	200	500	500
FBA/INST MFA	INST-MFA	FBA	INST MFA	INST MFA	INST MFA	INST MFA	INST MFA	INST MFA
Carboxylations								
Rubisco	116.29	122.38	166.40	170.06	213.35	119.24	129.33	117.53
PEP C	21.45	7.48	118.86	34.21	16.13	1.24	0.65	0.86
Decarboxylations								
ME	14.84	4.07	110.59	30.19	6.91			
PDH	12.32	17.68	20.67	16.10	29.95	0.79	0.51	0.62
ICDH	3.20	2.38	17.57	13.08	15.21	0.79	0.51	0.62
AKG	0.00	0.24	13.44	11.07	7.83	0.00	0.00	0.00
AA synthesis/No PDH-no ICDH	7.39	5.50	9.56	4.65	17.04	1.84	1.18	1.44
6PG	0.00	0.00	13.44	29.18	52.53			3.59
Photorespiration	0.00	0.00	0.00	0.00	0.00	17.05	27.77	12.10
Additional metabolic fluxes								
Glutamate formation = isocitrate – AKG	3.20	2.14	4.13	2.01	7.37	0.79	0.51	0.62
Lipid decarboxylation = PDH - ICDH	9.13	15.30	3.10	3.02	14.75	0.00	0.00	0.00
Total AA synthesis decarboxylations	13.78	10.25	9.56	4.65	17.04	1.84	1.18	1.44
Cellular carbon fluxes								
Gross C assimilation	137.74	129.86	285.26	204.27	229.48	120.47	129.98	118.39
Total decarboxylation	37.74	29.86	185.26	104.27	129.48	20.47	29.98	18.39
Net C assimilation	100.00	100.00	100.00	100.00	100.00	100.00	100.00	100.00
NU-decarboxylation rate	22.91	25.79	61.24	44.90	70.04	3.43	2.21	2.69
NU-decarbox rate/Net C assimilation	0.23	0.26	0.61	0.45	0.70	0.034	0.022	0.027

*Notes*: Results for all 11 open TCA cycle experiments are tabulated in the Supplemental Table S1. All fluxes are normalized to net assimilation = 100. RuBisCo is the rate of CO_2_ fixation by RuBisCo, and PEP C is the rate of CO_2_ fixation by PEP carboxylase. ME is the rate of decarboxylation by ME, PDH is decarboxylation of PYR, ICDH is decarboxylation of isocitrate, AKG is decarboxylation of AKG, 6-PG is decarboxylation of 6-PG, and photorespiration is CO_2_ release by photorespiration. “Lipid decarboxylations” correspond to decarboxylations by PDH in excess of isocitrate decarboxylations. “Amino acid synthesis decarboxylations” refer to decarboxylations associated with the synthesis of proteinogenic amino acids beyond decarboxylations associated with PDH or the TCA cycle.

Over the past decade, multiple papers have used FBA and INST-MFA to estimate metabolic fluxes in photoautotrophs. To measure fluxes by INST-MFA, one adds ^13^C-labeled HCO_3_^−^ to a culture at steady state, and then observes the progressive incorporation of ^13^C in various substrates as a function of time (Young et al., 2011; Ma et al., 2017; Babele and Young, 2020; Xu et al., 2021). In INST-MFA, the rate terms are fixed at values giving the best agreement between measured values of ^13^C in the metabolites, and values simulated by a model. The computed fluxes are therefore experimental observations. A characteristic of these studies is that they apply only to phytoplankton and plants grown at steady-state under specific conditions. When there are large changes in nutrient limitation, irradiance, or other environmental properties, rates will change, and metabolic pathways may be realized that are absent in the steady state (Turpin et al., 1988).

## Results

### FBA and INST-MFA studies quantify process-specific decarboxylation rates for a fully open TCA cycle

**Figure 3:**
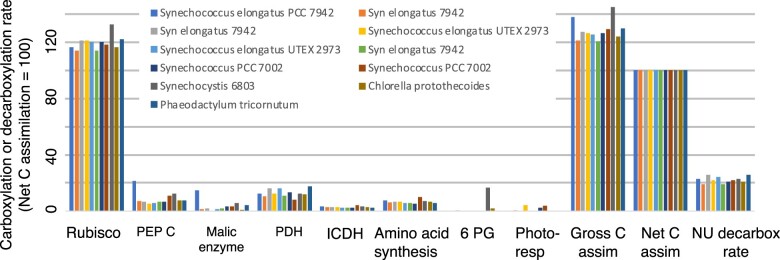
Bar diagram illustrating the decarboxylation fluxes associated with different processes in the intermediate metabolism when the TCA cycle is completely open. These fluxes were determined from FBA and INST-MFA studies where the AKG decarboxylation rate = zero (Table 2 and Supplemental Table S1). *Y*-axis values correspond to carbon fluxes normalized to a value of 100 for net C assimilation. “Rubisco” and “PEP C” refer to C fixation by these enzymes. “PDH,” “ICDH,” “6-PG”, and “Photoresp” (photorespiration) refer to the rate of decarboxylations associated with each of those enzymes or substrates. “NU-decarboxylations” are nonphotorespiratory decarboxylations uncompensated by carboxylations in a related metabolic pathway. Equations used to calculate gross and net C assimilation are given in the text. Data are summarized in Supplemental Table S1.

Rates in the intermediary metabolism for all experiments included in this study are summarized in Table 2, Figure 3 and Supplemental Table S1.

Decarboxylation of one PYR molecule produces one molecule of CO_2_ and one molecule of acetyl Co-A. Most acetyl Co-A either elongates a fatty acid chain or combines with OAA to enter the TCA cycle as citrate. The rate at which acetyl co-A combines with OAA equals the decarboxylation rate of isocitrate. This rate, based on results for 11 open-TCA cycle experiments, averages 2.81 isocitrate decarboxylations per 100 net C assimilation (see examples in Table 2, and the full data set in Supplemental Table S1, documented in Supplemental Text S4). The rate of all decarboxylations associated with amino acid synthesis is 4.31 times the isocitrate decarboxylation rate. One part of 4.31 parts comes from PYR decarboxylation, one part from isocitrate decarboxylation, and 2.31 parts come from decarboxylations in amino acid synthesis pathways outside the TCA cycle (see Supplemental Table S2, documented in Supplemental Text S5). There are no decarboxylations associated with ${\text{NH}}_{4}^{+}$ assimilation itself (Falkowski and Raven, 2007). For the completely open TCA cycle experiments, the average decarboxylation rate associated with amino acid synthesis is 2.81 * 4.31 = 12.1 CO_2_ decarboxylations, after fluxes are normalized to a value of 100 for net C assimilation. In the open TCA cycle, isocitrate decarboxylation to AKG provides carbon skeletons for the synthesis of glutamate, glutamine, proline, and arginine.

NU-decarboxylation associated with lipid synthesis derives from PDH. The decarboxylation rate of PYR to support lipid synthesis equals the PYR decarboxylation rate minus the isocitrate decarboxylation rate. In the 11 experiments where the TCA cycle is completely open, this difference averages 10.1 (Supplemental Table S1). Thus, in these samples, amino acid synthesis and lipid synthesis are responsible for similar rates of decarboxylations (12.1 and 10.1) in the light, normalized to net assimilation = 100. The NU-decarboxylation rate for the open-TCA cycle samples is 12.1 + 10.1 = 22.2, or 22% of net C assimilation. When the TCA cycle is open, only ∼ 2.81/22.2 = 13% of decarboxylations occur within the TCA cycle. Tcherkez et al. (2017b) previously concluded that plants also have low rates of TCA-cycle decarboxylations in the light. About 30% of decarboxylations originate in the amino acid synthesis pathways outside of PDH and the TCA cycle.

### Decarboxylation rates are higher when the TCA cycle is partly closed

Three experiments show a partly closed TCA cycle in the light, as reflected in the non-zero decarboxylation rate of AKG (Table 2). Cyanobacteria in these three experiments tend to have high carboxylation rates via PEP C and high decarboxylation rates due to ME. ME and PEP C fluxes are similar, after allowing for net CO_2_ uptake by the anapleurotic process, corresponding to a compensated link, as discussed earlier (and in Figure 2). All three partly closed experiments also show high decarboxylation rates of 6PG, which we treat as a compensated flux balanced by excess C fixation rates at RuBisCo.

**Figure 4:**
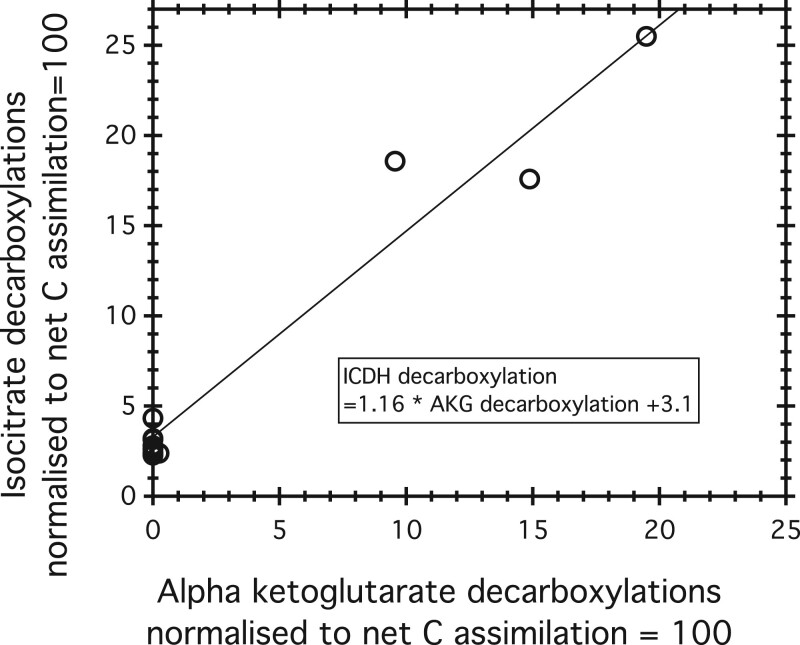
AKG decarboxylation rate versus isocitrate decarboxylation rate. The points fall along a line with a slope close to 1, but the data are scattered and few. The *y*-axis intercept corresponds to an isocitrate decarboxylation rate of 3.1 when AKG decarboxylation is zero and the TCA pathway is completely open. The remaining flux of isocitrate to AKG is decarboxylated, from AKG to succinate, in a partly closed TCA cycle.

The three partly closed experiments have very high rates of isocitrate decarboxylation, up to 18% of net assimilation (Table 2). In comparison, the average value of isocitrate decarboxylation is 2.8 in the experiments with the completely open TCA cycle, including a high value of 4.3, normalized to net CO_2_ assimilation = 100. When the TCA cycle is partly closed, isocitrate decarboxylations must rise to supply AKG for decarboxylation to succinate as well as for amination to glutamate. As a consequence, we expect isocitrate decarboxylation rates to increase linearly, with a slope of 1, with AKG decarboxylation rates. This relationship is more or less observed (Figure 4), but with few data and considerable scattering.

Subtracting the AKG decarboxylation rate from the isocitrate decarboxylation rate gives the flux of AKG to glutamate (Table 2). In the partly closed samples, the rate of glutamate production ranges from 2.0 to 7.4 (*n* = 3). Two values are within the range observed in open system experiments; one is much higher.

We can explain rates of processes in the partly closed TCA cycle as a consequence of open TCA cycle processes with four additions: (1) High decarboxylation rates by ME are linked to high carboxylation rates by PEPC. (2) High rates of 6 PG decarboxylation are, in our approach, associated with high carboxylation rates by RuBisCo. (3) High rates of isocitrate decarboxylation in partly closed experiments reflect the elevated isocitrate flux required to supply AKG for amination to glutamate as well as for decarboxylation to succinate. (4) High normalized rates of NU-decarboxylation are linked to high decarboxylation rates of PYR, isocitrate, and AKG in the partly closed samples.

### Cellular decarboxylation rates, and NU-decarboxylation as a fraction of net C assimilation, are constrained by the relative abundance of compound classes

Finkel et al. (2016) summarized the abundance of protein, lipids, carbohydrates, DNA, RNA, and chlorophyll in seven phytoplankton taxa. NU-decarboxylation/net C assimilation for these taxa, calculated, as outlined in “Materials and methods,” from their protein–lipid–carbohydrate abundances, are summarized in Table 3. The ratio of NU-decarboxylation/net assimilation ranges from 0.24 to 0.32, averaging 0.29. This range is similar to, although somewhat higher than, values obtained using FBA and INST-MFA (0.22). Similar values are calculated from other studies reporting compound class abundances (Parsons et al., 1961; Jonasdottir, 2019; Liefer et al., 2019); see Supplemental Table S3, documented in Supplemental Text S6.

**Table 3:** The calculation of NU-decarboxylations, normalized to net production = 100, from compound class abundance

Finkel et al. (2016)			
	Protein	Carbs	Lipids
(a) Fractional concentration; protein + lipids + carbs = 1.00			
Cyanobacteria	0.563	0.285	0.153
Chlorophyta	0.516	0.227	0.257
Cryptophyta	0.574	0.186	0.240
Bacillariophyta	0.469	0.209	0.322
Haptophyta	0.475	0.250	0.275
Ochrophyta	0.477	0.211	0.312
Dinophyta	0.414	0.347	0.239
(b) Net C assimilation/100 g of compound class			
	3.775	3.33	7.143
(c) Net C assimilation by compound class/100 g of biomass			
Cyanobacteria	2.124	0.948	1.091
Chlorophyta	1.947	0.756	1.836
Cryptophyta	2.166	0.620	1.714
Bacillariophyta	1.771	0.696	2.299
Haptophyta	1.793	0.833	1.965
Ochrophyta	1.802	0.702	2.228
Dinophyta	1.562	1.157	1.705
(d) Decarboxylations/100 g of compound class			
	0.803	0.000	3.572
(e) Decarboxylations by compound class /100 g of biomass			
Cyanobacteria	0.452	0.000	0.546
Chlorophyta	0.414	0.000	0.918
Cryptophyta	0.461	0.000	0.857
Bacillariophyta	0.377	0.000	1.150
Haptophyta	0.381	0.000	0.983
Ochrophyta	0.383	0.000	1.114
Dinophyta	0.332	0.000	0.853
(f) NU-decarboxylations/net C assimilation			
Cyanobacteria	0.24		
Chlorophyta	0.29		
Cryptophyta	0.29		
Bacillariophyta	0.32		
Haptophyta	0.30		
Ochrophyta	0.32		
Dinophyta	0.27		

*Notes*: Numbers in each cell indicate values of protein, carbohydrates, and lipids for the phytoplankton groups listed on the left. (a) “Fractional concentration” is the fraction of a compound class, by mass, in a sample of biomass. The fractional concentrations of proteins, lipids, and carbohydrates sum to 1.000. Data from Finkel et al. (2016). (b) “Net C production/100 g of compound class” gives the number of moles of C in 100 g of protein, carbohydrates, or lipids. (c) “Net production by compound class” gives the number of moles of C contributed by each compound class in 100 g of biomass. (d) “Decarboxylations/100 g of compound class” gives the number of decarboxylations required for the synthesis of 100 g of protein, carbohydrates, or lipids. (e) “Decarboxylations by compound class/100 g of biomass” gives the number of decarboxylations required to form the amount of biomass in 100 g of each compound class, according to standard biosynthetic pathways. (f) “NU-decarboxylations/net production” is the ratio of NU-decarboxylations required to forms 100 g of biomass, normalized to net production.

## Discussion

NU-decarboxylation/net C assimilation calculated from compound class abundance scatters around 0.25, with a high average of 0.29 from data of Finkel and collaborators. The ratio of NU-decarboxylation/net C assimilation computed from FBA and INST-MFA data is about 10% lower. Thus, two independent approaches support a value of about 0.25 ± 0.04 for the average ratio of NU-decarboxylation/net assimilation by algae and cyanobacteria. In both the FBA/INST-MFA and compound class analyses, cyanobacteria tend to have lower lipid concentrations than algae, explaining the lower ratios of NU-carboxylation/net C assimilation observed for cyanobacteria. In the case of the samples with completely open TCA pathways, the NU-decarboxylation rate was 22 ± 2% of net assimilation as calculated in FBA and INST-MFA studies. Of these 22 units, ∼10 corresponded to PYR decarboxylations producing fatty acids. Three units corresponded to PYR decarboxylations producing acetyl Co-A and combining with oxaloacetate to form citrate. The citrate produced in this way enters the TCA pathway, where it is decarboxylated to AKG (with three decarboxylations) and used for the synthesis of amino acids. Approximately six decarboxylations originated from amino acid synthesis outside PDH or the TCA cycle.

The approach and results outlined here have implications for several issues in the carbon cycle. Here, we highlight three. First, they enable an estimate of photoautotrophic decarboxylation rates, in the light, in aquatic ecosystems. In natural waters, the three dominant carbon fluxes are photosynthesis, autotrophic respiration, and heterotrophic respiration. Net rates of ecosystem carbon assimilation can often be readily measured. FBA and INST MFA studies, together with compound class analysis, enable estimates of autotrophic respiration of aquatic ecosystems in the light.

Secondly, in leaves, in the light, the rate of nonphotorespiratory decarboxylation is typically 3%–5% of net CO_2_ assimilation (Crous et al., 2017), compared with our estimate of ∼ 25% in phytoplankton. In the context of this paper, a simple explanation for this result is that carbohydrates, which are largely synthesized without NU-decarboxylations, are the dominant compound class of plants (Khalil et al., 2006; Pallardy, 2010). Lipids typically constitute a few percent of leaf biomass (Ekman et al., 2007; Yu et al., 2018), and the high C/N ratio of leaves reflects their low protein content. The respiration rate of phytoplankton in the light is much greater than that of plants because phytoplankton synthesize metabolites whose pathways involve many more decarboxylations than the biochemicals synthesized by plants. Results from two INST-MFA studies involving plants show that low non-photorespiratory respiration rates in plants are linked to lower synthesis rates of amino acids and lipids and more synthesis of carbohydrates (Ma et al., 2014; Xu et al., 2021; Table 2).

Finally, this work prompts a simple speculation about the origin of the Kok effect (the decrease in quantum yield of net assimilation with increasing irradiance at low values of irradiance [Kok, 1949; Tcherkez et al., 2017a]). We suggest that the Kok effect represents a change in decarboxylation rates as cells of photoautotrophs transition from the closed TCA cycle in the dark to the open TCA cycle in the light. Even if the rate of PYR decarboxylation were constant, there would be a change in the cellular decarboxylation rate during the light-to-dark transition, as AKG would be decarboxylated rather than aminated. Of course, there may be other changes in the intermediate metabolism affecting decarboxylation rates, which we cannot quantify. These changes might cause respiration rates to be either higher or lower in the light than in the dark, as observed by Crous et al. (2017) (and see the summary of Tcherkez and Atkin, 2021). We thus correctly predict that the respiration rate, or quantum yield of net assimilation, will likely change during the transition from the closed to the open TCA cycle, but cannot predict the direction of the change.

We can summarize the question of algal and cyanobacterial decarboxylation, in the light, in the following way. Photosynthetic efficiency and irradiance determine the gross rate at which new biomass forms. Enzymatic processes control the commitment of that biomass to proteins, lipids, carbohydrates, and less abundant components. Reaction stoichiometry then dictates the decarboxylation rates along the individual nonphotorespiratory and noncompensating pathways. These rates sum to the net rate of NU-decarboxylations in the light. In most FBA studies and INST-MFA experiments, the rate of NU-decarboxylation in the light is the same as the total rate of cellular respiration. The rate of NU-decarboxylation is on the order of 25% of net C assimilation.

## Materials and methods

### We can extract rates of reactions in the intermediate metabolism from results of FBA and INST-MFA studies

We collected rates of carboxylation and decarboxylation from published FBA and INST-MFA studies. From these values, we calculated NU-decarboxylation rates using relationships described below. For the most part, we consider only studies involving algae and cyanobacteria in the light. We restricted our analysis to wild type cells. Much of our approach follows recent work of Xu et al (2021) and Sweetlove et al. (2013).

We start with published results from FBA and INST-MFA studies summarized in Table 2, Figure 3, and Supplemental Table S1 (Young et al., 2011; Ma et al., 2014; Wu et al., 2015; Kim et al., 2016; Abernathy et al., 2017, 2019; Hendry et al., 2017; Jazmin et al., 2017; Nakajima et al., 2017; Qian et al., 2018; Broddrick et al., 2019; Xu et al., 2021). These papers report fluxes of the major carboxylation and decarboxylation reactions associated with carbon fixation and the synthesis of amino acids (or proteins), carbohydrates, and lipids. Where flux calculations were made from both the FBA and INST-MFA studies, we adopted the results from INST-MFA, as these are derived directly from measurements. Where FBA and INST-MFA experiments were done at different irradiances, we include both FBA and INST-MFA results. Calculations are normalized to a rate of net C assimilation = 100. This normalization puts all fluxes in the same units and facilitates comparisons.

The data set includes 11 cyanobacterial and microalgal experiments with zero decarboxylation fluxes of AKG, reflecting completely open-system conditions (Supplementary Table S1). Fluxes from two representative experiments are summarized in Table 2, and fluxes for all 11 experiments are presented in Supplementary Table S1. Fluxes from 2 of the 11 completely open experiments involve important CO_2_ sources from photorespiration. We also tabulate rates for three additional cyanobacterial experiments with important decarboxylation fluxes of AKG, indicating partly closed system conditions (Table 2 and Supplementary Table S1). Finally, we tabulate rates for three plant experiments as well (Table 2 and Supplementary Table S1).

We sum the NU-decarboxylation rates of the individual metabolites, giving the cellular decarboxylation rate in the light, again normalized to net CO_2_ assimilation = 100. The basic mass balance equations are as follows:

(2) Gross C assimilation=RuBisCo fixation+PEPC fixation.

(3) Total decarboxylation=ME+PDH+ICDH+amino acid synthesis decarboxylations outside the TCA cycle+OPPP+AKG+photorespiration.

(4) $${\text{Net CO}}_{2}\text{assimilation}\;\left(\text{or net assimilation}\right)=\text{Gross}\;C\;\text{assimilation}\;–\;\text{total decarboxylation}.$$

(5) NU−decarboxylation=PDH+ICDH+additional decarboxylations for amino acid synthesis+AKG decarboxylations.

The TCA cycle is assumed to be open when AKG decarboxylations ∼ 0.

### The rate of respiration in the light is constrained by the abundance of the different compound classes

In this alternative approach, we start with the fact that biomass equals net assimilation by definition. According to FBA and INST-MFA results above, the rate of NU-decarboxylation in the open TCA cycle is the sum of the decarboxylation rates required for the biosynthesis of each compound class. Knowing net assimilation, the NU-decarboxylation rate, and the composition of biomass, we can calculate the ratio of NU-decarboxylation to net assimilation.

We begin by calculating the abundance of compound classes in units of moles of C/100 g of compound (Table 1). This calculation is straightforward where the molecular weight of a compound is well defined. However, it requires assumptions about biomass composition when multiple compounds make up the compound class (e.g. the amino acids comprising protein: Supplemental Table S2). In Table 3 and Supplemental Table S3, we summarize measurements of the composition of each compound class. As stated above, we calculate respiration rates in the light based on the relative abundance of amino acids, lipids, and carbohydrates. In addition, invoking data of Liefer et al. (2019) we repeat some calculations including ribonucleic acids, chlorophyll, and accessory pigments, to evaluate the error associated with neglecting these biosyntheses (see Supplemental Table S3).

Based on standard metabolic pathways, we compute the number of decarboxylations attending the synthesis of 100 g of a given compound or compound class (Table 1). We then multiply the number of decarboxylations required to produce 100 g of the compound by the fraction of biomass composed of the given compound. Summing the decarboxylations from the different compounds or compound classes gives the total number of NU-decarboxylations required to produce 100 g of biomass. Similarly, one can calculate the amount of C in 100 g of biomass (i.e. net C assimilation). From these two numbers, we can then calculate the ratio of NU-decarboxylations/net C assimilation.

Above, we calculated the number of decarboxylations that will accompany the production of average phytoplankton biomass as estimated from the compilation of Jonasdottir (2019). In this calculation, we start with the fraction of protein, carbohydrate, and lipid in biomass. We know the number of decarboxylations per unit of biomass (Table 1). From these data, we can calculate the decarboxylation rate normalized to 100 units of net C assimilation. The ratio of NU-decarboxylation to net C assimilation, 1.123 moles/4.320 moles, is 0.26. This value is similar to values obtained using FBA and INST-MFA as presented above (0.22 ± 0.02).

There are a number of assumptions implicit in one or both of our approaches:

1. All syntheses of major compound classes in phytoplankton take place in the light. This assumption obviously obtains for culture samples grown in continuous light. It is also likely to be a good assumption for carbohydrates and fatty acids when phytoplankton spend time in the dark, either in the field or in cultures with light–dark cycles (Lacour et al., 2012). However, the situation for amino acid syntheses is more complicated. Cuhel et al. (1984) measured rates to be similar in the light and the dark. Hama et al. (1987) (working in a lake) and Probyn et al. (1990) observed dark rates that were less than rates in the light, but still important. Kanda et al. (1989), Glover and Smith (1988), and Granum et al. (2002) suggest that N assimilation, and protein synthesis, is predominantly a light process. A simple calculation indicates the magnitude of error introduced into respiration rates in the light due to amino acid synthesis in the dark. Assume that the rate of amino acid synthesis is equal in the light and in the dark, and that the respiration rate is equal in the light and in the dark. Assume that half of NU-decarboxylations were due to amino acid synthesis and half to fatty acid synthesis. In this case, the correct normalized NU-decarboxylation rate in the light would be lower, by 25%, than the rate we calculate. Citations above suggest that the real error is much smaller.
2. In doing compound class analyses, we assume AKG is aminated rather than carboxylated (i.e. the TCA cycle is completely open in the light). Based on FBA and INST-MFA studies summarized here, this assumption is generally correct but not always (see Table 2 and Supplemental Table S1). When the TCA cycle is partly closed, decarboxylations normalized to net assimilation must be higher than when the TCA cycle is fully open.
3. The stoichiometry is properly represented. In fact, there are inevitable uncertainties when describing the synthesis of a compound class such as protein, lipids, or accessory pigments, where many different compounds are involved.
4. The abundance of pigments, ribonucleic acids, and other compounds is low enough that they can be neglected without introducing large errors into the calculation of NU-decarboxylations based on compound class analysis. Including the carbon burden and decarboxylation requirements of chlorophyll, accessory pigments, and ribonucleic acids (Table 1) raises the calculated ratio of NU-decarboxylations/net assimilation by about 2% of the ratio (e.g. from 25.0% to 25.5%). This sensitivity is calculated based on cell composition data of Liefer et al. (2019); see Supplemental Table S3. In general, omitting compounds other than proteins, lipids, and carbohydrates is associated with a very small error in the ratio of NU-decarboxylations/net assimilation, and other rate terms evaluated in this paper.
5. Proteins and lipids are stable or, if degraded, do not undergo decarboxylations. Under severe stress, these compounds can be degraded to CO_2_ in order to release energy. In addition, lipids and starch may be degraded in the dark to produce energy for nighttime respiration as part of the diurnal cycle of healthy cells (Becker et al., 2018). Here, we examine whether or not the degradation of proteins and lipids in the light, by healthy cells, would lead to substantial rates of decarboxylation. Regarding proteins, median degradation rates reported for Arabidopsis and algae are typically ∼ 0.1–0.2 day^−1^ (Quigg and Beardall, 2003; Aryal et al., 2012; Martin et al., 2012). Of course, protein degradation rates are highly variable. For example, rates for proteins associated with the light and dark reactions of photosynthesis can be much faster (Mastrobuoni et al., 2012; Yao et al., 2012; Nelson et al., 2014). On the other hand, Karlsen et al. (2021) emphasized the slowness of protein turnover and the conservation of the proteome. Given phytoplankton doubling times of order 1 day^−1^, most synthesized protein must be committed to growth.  The products of protein degradation are amino acids, which are produced without decarboxylation. Amino acids can either be reassimilated or further degraded. Amino acid degradation takes place along standard pathways until substrates are produced that can enter into gluconeogenesis or be transformed to other useful metabolites (Voet and Voet, 2011; Sawers, 2015). Some amino acids are decarboxylated along these standard pathways. We calculated these decarboxylation rates using the same approach as for calculating decarboxylations associated with amino acid synthesis (Supplemental Table S2). The overall rate is 0.3 amino acid decarboxylations per 100 g of amino acids, for the extreme case where all amino acids are degraded to their terminal substrates. In comparison, there are 0.8 amino acid decarboxylations per 100 g of amino acids assimilated, in addition to requisite decarboxylations at PDH and ICDH. Overall, protein degradation is slow, and the decarboxylations rate associated with amino acid degradation is small. Therefore, decarboxylation linked to protein and amino acid degradation is a small fraction of cellular decarboxylation.  In the light, lipids are degraded in two steps (Terashima, 2017; Kong et al., 2018). The first is lipolysis, which cleaves the head groups from the acyl chains. The second is beta-oxidation, which shortens the acyl chains while producing acetyl co-A. Acetyl co-A will generally enter biosynthetic pathways. These processes of lipid and fatty acid degradation lead to little or no decarboxylation. Support for this conclusion comes from the recent study of Young and Shachar-Hill (2021). Through ^14^C labeling experiments, they showed that there was no detectable loss of lipid carbon as *Chlamydomonas reinhardtii* cells cycled between N-limiting and N-replete conditions. This high retention occurred despite large variations in lipid composition, with membrane lipids dominating in N-sufficient conditions, and triacylglycerol dominating in N-limited conditions. Retention of the ^14^C label thus suggests low levels of decarboxylation due to lipid degradation in the light. Again, lipid degradation in the dark may be part of the diurnal cycle of healthy cells.

## Supplemental data

**Supplemental Table S1.** FBA + INST-MFA results: calculating the net number of decarboxylations associated with the synthesis of amino acids, lipids, chlorophyll, carotenoids, and ribonucleic acids.

**Supplemental Table S2.** Amino acid decarboxylations: calculation of decarboxylations associated with the syntheses of the proteinogenic amino acids.

**Supplemental Table S3.** Compound class analysis: calculation of NU-decarboxylation rate, normalized to net carbon assimilation, based on the abundance of amino acids, lipids, and carbohydrates in phytoplankton.

**Supplemental Text S1.** List of abbreviations.

**Supplemental Text S2.** Calculating the number of decarboxylations required for the synthesis of the individual components of biomass: protein, lipids, carbohydrates, chlorophyll, accessory pigments (carotenoids), and DNA+RNA.

**Supplemental Text S3.** Calculating the number of moles of C required to produce 100 grams of each synthesized compound or compound class.

**Supplemental Text S4.** Documentation for Supplemental Table S1, FBA + INST-MFA results.

**Supplemental Text S5.** Documentation for Supplemental Table S2, amino acid decarboxylation.

**Supplemental Text S6.** Documentation for Supplemental Table S3, compound class analysis.

## Supplementary Material

kiac254_Supplementary_Data

## Notes

M.L.B. designed the research, performed the research, analyzed the data, and took the lead in writing the paper. X.-G.Z., P.F., F.M., and K.G. performed the research, analyzed the data, and contributed to the writing of the paper.

The author responsible for distribution of materials integral to the findings presented in this article in accordance with the policy described in the Instructions for Authors (https://academic.oup.com/plphys/pages/general-instructions) is: Michael L. Bender (bender@princeton.edu).
