# Supplementary material for: On the rate of phytoplankton respiration in the light
Source: Plant Physiol. 2022 Jun 2;190(1):267–79. doi: 10.1093/plphys/kiac254 (PMC9434318; doi:10.1093/plphys/kiac254)
Supplement: kiac254_Supplementary_Data [file kiac254_supplementary_data.zip › 00151supptext.pdf]

On the rate of phytoplankton respiration in the light: Supplementary online material

The Supplementary Material consists of an Excel workbook with 3 worksheets, and a single pdf (this file) with 6 text sections.

The contents of the Excel Worksheet are:

Supplemental Table S1: FBA + INST-MFA results: Calculating the net number of decarboxylations associated with the synthesis of amino acids, lipids, chlorophyll, carotenoids, and ribonucleic acids

Supplemental Table S2: Amino acid decarboxylations: Calculation of decarboxylations associated with the syntheses of the proteinogenic amino acids. Also included: calculation of decarboxylations associated with the degradation of proteinogenic amino acids to substrates that re-enter the biosynthesis pathways.

Supplemental Table S3: Compound class analysis: Calculation of NU-decarboxylation rate, normalized to net carbon assimilation, based on the abundance of amino acids, lipids, and carbohydrates in phytoplankton. Also calculated are synthesis decarboxylation rates involving 3 additional substrates (chlorophyll, accessory pigments, and ribonucleic acids).

The contents of the pdf file are:

Supplemental Text S1. List of abbreviations.

Supplemental Text S2. Calculating the number of decarboxylations required for the synthesis of the individual components of biomass: Protein, lipids, carbohydrates, chlorophyll, accessory pigments (carotenoids), and DNA+RNA.

Supplemental Text S3. Calculating the number of moles of C required to produce 100 grams of each synthesized compound or compound class .

Supplemental Text S4. Documentation for Excel Workbook Tab 1, FBA + INST-MFA results.

Supplemental Text S5. Documentation for Excel Workbook Tab 2, amino acid decarboxylation.

Supplemental Text S6. Documentation for Excel Workbook Tab 3, Compound class analysis.

Supplemental Text S1. List of abbreviations

6PG	6 phosphoglucanate
AcCoA	acetyl coA
AKG	alpha ketoglutarate
AKGDH	alpha keotglutarate dehydrogenase
Cit	Citrate
F6P	fructose-6-phosphate
FBA	flux balance analysis
FBP	fructose 1-6 bisphosphate
Fum	fumarate
G6P	glucose-6-phosphate
GAP	glyceraldehyde-3-phosphate
ICDH	isocitrate dehydrogenase
INST-MFA	Isotopically nonstationary mass flux analysis
Isocit	isocitrate
Mal	malate
ME	malic enzyme
NU-decarboxylation	nonphotorespiratory, uncompensated decarboxylation
OAA	oxaloacetate
OPPP	oxidative pentose phosphate pathway
PDH	pyruvate dehydrogenase
PEP	phosphoenolpyruvate
Pep C	phosphoenolpyruvate carboxylase
PGA	Phosphoglycerate
Pyr	pyruvate
R5P	ribose 5 phosphate
RuBP	riulose 1-5-bisphosphate
TCA cycle	tricarboxylic acid cycle
TP	triose phosphate

Supplemental Text S2. Calculating the number of decarboxylations required for the synthesis of the individual components of biomass: Protein, lipids, carbohydrates, chlorophyll, accessory pigments (carotenoids), and DNA+RNA

1. Decarboxylations linked to amino acid synthesis

Our calculation of decarboxylation rates associated with the formation of amino acids, or proteins, involve the following steps or observations:

(a) The synthesis of glutamate, glutamine, arginine and proline molecules involve one decarboxylation at PDH and a second at isocitrate dehydrogenase (ICDH). Syntheses of the proteinogenic amino acid do not involve any other decarboxylations at PDH or in the TCA cycle.

(b) The synthesis of each proteinogenic amino acid involves a starting substrate that is produced from 3 phosphoglycerate, usually without uncompensated carboxylations or decarboxylations.

(c) Standard metabolic pathways (Voet and Voet, 2011) specify the number of carboxylations and decarboxylations involved in the formation of each amino acid from its starting substrate.

(d) The formation rate of each amino acid is proportional to its abundance in algal protein as observed by (Brown, 1991).

From this information, and the fraction of protein in biomass, we can calculate the number of uncompensated decarboxylations associated with the production of the proteinogenic amino acids outside of PDH and the TCA cycle. This number is 2.31 times the rate of isocitrate decarboxylation. There are of course additional decarboxylations at PDH and in the TCA cycle associated with the synthesis of glutamate, glutamine, arginine, and proline. The product is the number of decarboxylations associated with production of protein, normalized to net C assimilation = 100. Details are outlined in the Supplementary Excel file, Tab 2, “Amino acid decarboxylations”.

We note that most synthesis decarboxylations are associated with a small number of the proteinogenic amino acids. Thus, there may be considerable variability in the uncompensated carboxylations and decarboxylations associated with amino acid synthesis.

2. Decarboxylations linked to lipids

We represent lipids as having the composition n-CH_2. This representation asserts that the composition of lipids is dominated by the fatty acid chains rather than the head groups. The source of the carbon in lipids is mainly the decarboxylation of pyruvate. The product is acetyl Co-A, which is then used for elongating the fatty acid chain (Rawsthorne, 2002; Xu and Shanklin, 2016). The resulting stoichiometry is that 1 molecule of CO_2 is produced via PDH for every 2 C atoms assimilated into fatty acids.

3. Decarboxylations linked to chlorophyll synthesis

In the chlorophyll synthesis pathway, there are 4 decarboxylations during the transformation of uroporphyrinogen to coproporphorinogen, and 2 more from coproporphorinogen to protoporphorinogen (Tripathy and Pattanayak, 2012). An additional 10 decarboxylations are required to produce the fatty acid tail of the chlorophyll molecule. Overall, the synthesis of 1 molecule of chlorophyll, with 55 C atoms, involves 16 decarboxylations.

4. Decarboxylations linked to carotenoids

We represent carotenoids as a pigment comprised of multiple units of isoprene (C_5H_8). There are 2 pathways of isoprene synthesis. The mevalonate pathway starts with 3 pyruvate molecules that are decarboxylated to produce 3 CO_2 and 3 acetyl Co-A molecules. The acetyl Co-A molecules are condensed to form a 6-C compound, which is decarboxylated to make 5-C isoprene. The mevalonate pathway thus involves 4 decarboxylations to make an isoprene molecule with 5 C atoms. The 2-C-methyl-D-erithretol 4-phosphate (MEP) pathway of isoprene synthesis begins with the condensation of pyruvate and glyceraldehde 3 phosphate to form a 6-C molecule. A single decarboxylation, plus a molecular rearrangement, lead to the formation of isoprene. In the MEP pathway, there is a single decarboxylation to form one molecule of

isoprene. The MEP pathway dominates in phytoplankton (Xu and Shanklin, 2016). We therefore invoke MEP stoichiometry to calculate decarboxylations associated with carotenoid synthesis.

5. Decarboxylations linked to the production of DNA and RNA

The syntheses of adenine and guanine (Voet and Voet, 2011) involve a decarboxylation of 6-phosphogluconate to form Ru5P in the pentose phosphate pathway. Ru5P is transformed into R5P, which is the starting point for the synthesis of adenine and guanine. There is a carboxylation at step 7 of the synthesis (Voet and Voet, 2011) (p. 1109), and no other carboxylations or decarboxylations. Thus, there are no net decarboxylations in the synthesis of adenine and guanine.

Leading to the formation of cytosine and thymine, there is a carboxylation in the first step of the synthesis of uridine monophosphate, and a decarboxylation at the last step. There is also a decarboxylation associated with the addition of R5P, as for adenine and guanine. Thus, there is one net decarboxylation in the formation of each molecule of cytosine monophosphate or thymine monophosphate.

Based on these pathways, we have calculated the number of NN-decarboxylations depending on whether we consider biomass to include only proteins, carbohydrates, and lipids, or expand the definition to also include carotenoids, chlorophyll, and ribonucleic acids. As indicated in the text and Worksheet 3 in the Supplementary Workbook, including carotenoids, chlorophyll, and ribonucleic acids changes the ratio of NU-decarboxylation/net production by only about 2% of its value (i. e., 2% of the ratio of NU-decarboxylations/net C assimilation). We therefore exclude decarboxylations associated with the synthesis of carotenoids, chlorophyll, and ribonucleic acids from our analysis.

Supplemental Text S3. Calculating the number of moles of C required to produce 100 grams of each synthesized compound or compound class

Here we summarize the equations for calculating the number of moles of C, and the number of decav rboxylations, contributed by each compound class to 100 grams of biomass (SOM

Table 2; also see caption to Fig. 4 in the main text). First, consider the case where the composition of a compound class is represented by a single compound.

The number of moles (n) of a compound in 100 grams of that compound is:

n_{100} = *100 grams/molecular weight* SOM (1)

The number of moles of C in 100 grams of the compound is:

$N_{C\text{-}100} = n_{100}$ *x the number of C atoms/molecule* SOM (2)

The number of moles of C in a compound making up a fraction (f) of the total biomass is:

Moles C contributing to biomass = $n_{C\text{-}100}$ *x f* SOM (3)

Moles of C in 100 grams of biomass from multiple compounds = $n_{C\text{-}100}(1)$ *x f(1)* + $n_{C\text{-}100}(2)$ *x f(2)* + $n_{C\text{-}100}(3)$ *x f(3) +...* SOM (4)

The numbers in parentheses correspond to the first, second, third…compound.

If the compound class itself contains multiple compounds, as is the case for amino acids, then the weighted average composition of the compound class is calculated from equations analogous to those above.

The number of biosynthesis decarboxylations required to produce 100 grams of biomass is given by multiplying the molar abundance of each compound class per 100 grams times the number of decarboxylations required to produce 1 mole of that compound class:

Moles of C decarboxylated per 100 grams of biomass from multiple compounds = $n_{C\text{-}100}(1)$ *x f(1) x DN(1)* + $n_{C\text{-}100}(2)$ *x f(2) x DN2* + $n_{C\text{-}100}(3)$ *x f(3) x DN(3) +...* SOM (5)

DN is the number of moles of decarboxylations required for the synthesis of 1 mole of the relevant compound class.

The ratio of decarboxylations to net C assimilation equals the ratio of moles C decarboxylated to produce 100 grams of biomass to the number of moles of C in 100 grams of biomass. Gross C assimilation equals net C assimilation + the number of decarboxylations. Therefore, the decarboxylation rate / gross C production equals decarboxylation rate / (decarboxylation rate + net C production). The basic mass balance constraints invoked here are

similar to those invoked to calculate the normalized number of decarboxylations linked to amino acid synthesis

Supplemental Text S4. Documentation for Excel Workbook Tab 1, FBA + INST-MFA results: Process-specific carboxylation and decarboxylation rates computed from FBA studies or measured in INST-MFA experiments. Rows 2-5 give the citation and information about experimental conditions. Rows 9 and 10 give carboxylation rates for the anapleurotic process and for Rubisco. Rows 13-29 give fluxes at scales of the enzyme and whole cells. Row 31 gives decarboxylation rates normalized to net C assimilation = 100, and row 32 gives NU-decarboxylation rate as a fraction of net C assimilation.

Columns B-L give results for experiments in which the TCA cycle is fully open (AKG decarboxylations ~ zero). Columns M-O give results for experiments where the TCA cycle is partly closed (reflected in a significant decarboxylation flux of AKG). Columns P-R summarize results for 3 experiments with plants.

Citations: Young et al., 2011; Ma et al., 2014; Wu et al., 2015; Kim et al., 2016; Abernathy et al., 2017; Hendry et al., 2017; Jazmin et al., 2017; Nakajima et al., 2017; Qian et al., 2018; Abernathy et al., 2019; Broddrick et al., 2019; Xu et al., 2021.

Supplemental Text S5. Documentation for Excel Workbook Tab 2, amino acid decarboxylation: Calculation of the ratio of decarboxylations required for amino acid synthesis not involving PDH and isocitrate, normalized to decarboxylations of isocitrate when the TCA cycle is open.

Column A: the individual proteinogenic amino acids.

Columns C-R: abundance of amino acids in protein of 17 algal species as reported by (Brown, 1991). Asparagine was not reported and its abundance is assumed to be zero. Glutamine was not reported and its abundance was assumed to equal 0.234 x glutamate abundance based on the relative abundance of amino acids in plants reported by (Kumar et al., 2017).

Columns T-X: Number of C, H, N, O, and S atoms per molecule.

Column Z: Decarboxylations outside PDH and the TCA cycle in the synthesis pathway of each amino acid. These are determined from the standard pathways of amino acid synthesis as outlined in Voet and Voet.

Column AA-AB: Average weight percent of each amino acid, and standard deviation.

Columns AC-AF: Relative molar abundance of each amino acid in protein (AC), molar abundance of amino acid in protein (normalized to sum=100%) (AD), relative mass abundance of each amino acid in protein (AE), and number of moles of each amino acid in 100 grams of protein (AF).

Column AG: Starting substrate for the synthesis of each amino acid.

Column AH: Total number of decarboxylations at PDH and ICDH required for the synthesis of 1 molecule of amino acid.

Column AI: Total number of decarboxylations required for the synthesis of 1 molecule of amino acid outside PDH and ICDH (same as Column Z).

Column AJ: Moles of decarboxylations in the synthesis of each amino acid required to produce the amount of that amino acid in 100 grams of protein.

Column AK: Moles of C contributed by each amino acid to account for 100 grams of protein.

Column AM: Moles of N contributed by each amino acid to account for 100 grams of protein.

Cell AJ34: Number of moles of isocitrate decarboxylation.

Cell AJ36: Number of moles of C decarboxylations outside pyruvate and isocitrate decarboxylation.

Cell AJ31: Number of decarboxylations required to produce 100 gm of protein, assuming the relative amino acid abundances of Brown (1991).

Cell AJ34: Number of isocitrate decarboxylations required to produce 100 gm of protein, assuming the relative amino acid abundances of Brown (1991).

Cell AJ36: Number of decarboxylations excluding PDH and isocitrate required to produce 100 gm of protein, assuming the relative amino acid abundances of Brown (1991).

Cell AJ38: Ratio of decarboxylations excluding ICDH and PDH to decarboxylations of ICDH. Since ICDH decarboxylations are reported in FBA and INST-MFA studies, this ratio allows us to calculate the decarboxylation rate associated with amino acid synthesis outside PDH and the TCA cycle.

Columns AO-AR: Calculating decarboxylations required to produce intermediate degradation products (Column AP) from the proteinogenic amino acid listed in column AO. “Intermediate degradation products” refers to the formation of an intermediate that can reenter the biosynthetic pathways rather than being oxidized all the way to CO_2 and H_2O. Column AQ tabulates the number of decarboxylations to produce the degradation product from a parent amino acid molecule. Column AR is the number of decarboxylations required to produce intermediate degradation products from 100 grams of protein, given the abundance of that particular amino acid in 100 grams of protein (Column AF).

Cell AR31 is the sum of decarboxylations associated with the degradation of each amino acid in 100 grams of protein. In other words, it is the sum of decarboxylations for the case where 100 grams of amino acids, with relative abundances as in (Brown, 1991)are degraded to their intermediate degradation state. Cell AJ31 is the number of decarboxylations associated with synthesis rather than degradation. Comparing cell AR31 with cell AJ31 shows that, in the extreme (and impossible) case that all amino acids in protein were degraded to their terminal intermediate substrates, the ratio of degradation decarboxylations to synthesis decarboxylation would be 0.308/0.803 = 0.38.

Supplemental Text S6. Documentation for Excel Workbook Tab 3, Compound class analysis: Calculations of the NU-decarboxylation rates normalized to net carbon assimilation =1.000, for data sets of Parsons et al. (1961) Finkel et al., (2016), Jonasdottir (2019) and Liefer et al. (2019). As exemplified by data of Finkel et al. (2016) (columns A-D): cells 5B-11D give relative concentrations of protein, carbohydrates and lipids normalized to a sum of 1.000. Cells 18B-18D give net C assimilation (moles)/100 grams of compound class (i. e., for 100 grams of protein, carbohydrates, or lipids (Table 1). Cells B21-D27 give net C assimilation (moles)/100

grams of biomass. Cells A33-D34 give decarboxylation rates (moles)/100 grams of compound class (Table 1). Cells B36-D43 give decarboxylation rates (moles) by compound class/100 grams of biomass.

Columns P-S show identical calculations for data of Liefer et al. (2019). Columns U- AA show the calculation of NU-decarboxylation rates/net C assimilation calculated by adding data for chlorophyll, carotenoids, and ribonucleic acids to the data for proteins, carbohydrates and lipids. The ratio of NU-decarboxylation rates/net C assimilation for 6 compounds (cells V50-V57) is virtually identical to the ratio for 3 compounds (cells Q50-Q57).

References for papers cited in the Supplementary Texts

Abernathy M. H., Czajka J. J., Allen D. K., Hill N. C., Cameron J. C. and Tang Y. J. (2019) Cyanobacterial carboxysome mutant analysis reveals the influence of enzyme compartmentalization on cellular metabolism and metabolic network rigidity. *Metabolic Engineering* **54**, 222–231.

Abernathy M. H., Yu J., Ma F., Liberton M., Ungerer J., Hollinshead W. D., Gopalakrishnan S., He L., Maranas C. D., Pakrasi H. B., Allen D. K. and Tang Y. J. (2017) Deciphering cyanobacterial phenotypes for fast photoautotrophic growth via isotopically nonstationary metabolic flux analysis. *Biotechnology for Biofuels* **10**.

Broddrick J. T., Welkie D. G., Jallet D., Golden S. S., Peers G. and Palsson B. O. (2019) Predicting the metabolic capabilities of Synechococcus elongatus PCC 7942 adapted to different light regimes. *Metabolic Engineering* **52**, 42–56.

Brown M. R. (1991) The amino-acid and sugar composition of 16 species of microalgae used in mariculture. *Journal of Experimental Marine Biology and Ecology* **145**, 79–99.

Finkel Z. F., Follows M. J., Liefer J. D., Brown C. M., Benner I. and Irwin A. J. (2016) Phylogenetic diversity in the macromolecular composition of microalgae. *Public Library of Science One* **11**, 1–16.

Hendry J. I., Prasannan C., Ma F., Möllers K. B., Jaiswal D., Digmurti M., Allen D. K., Frigaard N.-U., Dasgupta S. and Wangikar P. P. (2017) Rerouting of carbon flux in a glycogen mutant of cyanobacteria assessed via isotopically non-stationary ^{13}C metabolic flux analysis: Rerouting of Carbon Flux in a Glycogen Mutant. *Biotechnol. Bioeng.* **114**, 2298–2308.

Jazmin L. J., Xu Y., Cheah Y. E., Adebiyi A. O., Johnson C. H. and Young J. D. (2017) Isotopically nonstationary ^{13}C flux analysis of cyanobacterial isobutyraldehyde production. *Metabolic Engineering* **42**, 9–18.

Jonasdottir S. (2019) Fatty Acid Profiles and Production in Marine Phytoplankton. *Marine Drugs* **17**, 151.

Kim J., Fabris M., Baart G., Kim M. K., Goossens A., Vyverman W., Falkowski P. G. and Lun D. S. (2016) Flux balance analysis of primary metabolism in the diatom *Phaeodactylum tricornutum*. *The Plant Journal* **85**, 161–176.

Kumar V., Sharma A., Kaur R., Thukral A. K., Bhardwaj R. and Ahmad P. (2017) Differential distribution of amino acids in plants. *Amino Acids* **49**, 821–869.

Liefer J. D., Garg A., Fyfe M. H., Irwin A. J., Benner I., Brown C. M., Follows M. J., Omta A. W. and Finkel Z. V. (2019) The Macromolecular Basis of Phytoplankton C:N:P Under Nitrogen Starvation. *Frontiers in Microbiology* **10**.

Ma F., Jazmin L. J., Young J. D. and Allen D. K. (2014) Isotopically nonstationary ^{13}C flux analysis of changes in *Arabidopsis thaliana* leaf metabolism due to high light acclimation. *Proc Natl Acad Sci USA* **111**, 16967–16972.

Nakajima T., Yoshikawa K., Toya Y., Matsuda F. and Shimizu H. (2017) Metabolic flux analysis of the Synechocystis sp. PCC 6803 ΔnrtABCD mutant reveals a mechaism for metabolic adaptation to nitrogen-limited conditions. *Plant and Cell Physiology* **58**, 537–545.

Parsons T. R., Stephens K. and Stickland J. D. H. (1961) On the chemical composition of marine phytoplankters. *Journal of the Fisheries Research Board of Canada* **18**, 1001–1015.

Qian X., Zhang Y., Lun D. S. and Dismukes G. C. (2018) Rerouting of Metabolism into Desired Cellular Products by Nutrient Stress: Fluxes Reveal the Selected Pathways in Cyanobacterial Photosynthesis. *ACS Synthetic Biology* **7**, 1465–1476.

Rawsthorne S. (2002) Carbon flux and fatty acid synthesis in plants. *Progress in Lipid Research* **41**, 182–196.

Tripathy B. C. and Pattanayak G. K. (2012) Chlorophyll biosynthesis in higher plants. In *Photosynthesis: plastid biology energy conversion and carbon assimilation* Advances in Photosynthesis and Respiration. Springer, Netherlands. pp. 63–94.

Voet D. and Voet J. G. (2011) *Biochemistry*. Fourth edition, John Wiley and Sons, Hoboken, NJ.

Wu C., Xiong W., Dai J. and Wu Q. (2015) Genome-Based Metabolic Mapping and 13C Flux Analysis Reveal Systematic Properties of an Oleaginous Microalga *Chlorella protothecoides*. *Plant Physiology* **167**, 586–599.

Xu C. and Shanklin J. (2016) Triacylglycerol Metabolism, Function, and Accumulation in Plant Vegetative Tissues. *Annual Review of Plant Biology* **67**, 179–206.

Xu Y., Fu X., Sharkey T. D., Shachar-Hill Y. and Walker, B. J. (2021) The metabolic origins of non-photorespiratory CO2 release during photosynthesis: a metabolic flux analysis. *Plant Physiology* **186**, 297–314.

Young J. D., Shastri A. A., Stephanopoulos G. and Morgan J. A. (2011) Mapping photoautotrophic metabolism with isotopically nonstationary 13C flux analysis. *Metabolic Engineering* **13**, 656–665.
